# THE STARTING A TESTOSTERONE AND EXERCISE PROGRAM AFTER HIP INJURY (STEP-HI) TRIAL: STUDY RESULTS AND IMPLICATIONS

**DOI:** 10.1093/geroni/igae098.0270

**Published:** 2024-12-31

**Authors:** Ellen Binder, Douglas Kiel

**Affiliations:** Chair; Discussant

## Abstract

Hip fractures are common among older women and can have a devastating impact on their ability to remain independent. Many women who were high functioning before the hip fracture do not return to their pre-fracture level of function, have persistent weakness and mobility impairments, and may require ongoing supportive services. Age-associated androgen deficiency may contribute to deficits in muscle mass, strength and power that are common in older female hip fracture patients. The typical approach to hip fracture rehabilitation routinely includes a limited course of physical therapy but the precise role of exercise and the use of an anabolic therapy like testosterone has not been determined. The Starting a Testosterone and Exercise Program after Hip Injury (STEP-HI) Study is a three-group, randomized, double-blinded, placebo-controlled Phase III clinical trial that was conducted at 8 clinical sites in the USA. The trial was designed to evaluate a multi-modal intervention aimed at improving functional outcomes in older female hip fracture patients with persistent mobility impairment. 129 female hip fracture patients, age 65 yrs. and older were randomly assigned to one of three groups: supervised exercise plus 1% testosterone topical gel (EX+T); supervised exercise plus placebo gel (EX+P); or Enhanced Usual Care (EUC). This symposium will present the results of the trial, including recruitment and retention, primary and secondary outcome measures. The program will also address strategies to ensure fidelity with exercise in a multicenter study like STEP-HI. Implications of study findings for future research and clinical practice will be discussed.

